# A Case for Cerebellar Neuromodulation in Affective Disorders

**DOI:** 10.1192/bjo.2024.680

**Published:** 2024-08-01

**Authors:** Alex O'Neill-Kerr, Harshani Yapa Bandara, Nadia Hristova

**Affiliations:** 1 Transforming Mind Solutions, Northampton, United Kingdom; 2 Northamptonshire Healthcare NHS Foundation Trust, Northampton, United Kingdom; 3 Bright Brain Center, London, United Kingdom

## Abstract

**Aims:**

This case report focuses on a 68-year-old Caucasian female, with long-standing symptoms of executive and cerebellar dysfunction, which responded well to rTMS targeting the prefrontal cortex and cerebellum.

**Methods:**

This patient was seen in the private sector for long-standing symptoms of low mood, mental fog, unsteady gait, along with slurred speech and poor vision. History indicated the possibility of multiple mini strokes several years earlier, which may have contributed to her current presentation, and MRI Brain confirmed diffuse small vessel disease in periventricular areas and deep white matter, with no atrophy of brainstem or cerebellum. Her medications at the time included venlafaxine 75 mg OD, atorvastatin 20 mg OD, amlodipine 5 mg OD, thiamine HCL 100 mg OD and aspirin 75 mg. She then had a course of standard rTMS (F3 and F4), to which she had no real response. The team then performed a Quantitative Electroencephalography which revealed bilateral prefrontal and cerebellar disconnection, with normal connectivity in the rest of the brain and cortex, which enabled a diagnosis of Organic mood (affective) disorder, F06.3.

Based on the above findings, she was then prescribed a course of rTMS as follows:

1. Bifrontal excitatory Theta burst, at 50–60%, daily for 20 treatments.
2. CB1 and CB2 Cerebellar iTBS (10min) at 50–60%, daily for 20 treatments.

Mid treatment review showed improvement in depression and anxiety, mirrored by significant reductions in psychometric scores compared to baseline, although her memory, concentration and motivation remained poor, and it was agreed that she would be reviewed again after completion of 20 sessions.

**Results:**

Although long known for its function in fine tuning motor function, emerging research indicates the growing importance of the cerebellum and its neural connections in neuropsychiatric disorders.

Recent studies have shown that those with cerebellar damage show impairments in executive function, and emotional regulation, in addition to language deficits and problems with sensory processing. It is bidirectionally connected with areas associated with processing social salience, including the posterior parietal and prefrontal cortex. With its connections to the prefrontal cortex, limbic structures and monoamine producing brainstem areas, it is likely the cerebellum also plays a key role in fine tuning emotional output, which appears to be corroborated by functional neuroimaging.

**Conclusion:**

This case further supports the emerging evidence base that the cerebellum plays a key role in emotional experience, along with the prospect of using targeted rTMS for therapeutic benefit.

